# Hydrogel Bead-Based Assay for the Measurement of Protein Biomarkers in Saliva

**DOI:** 10.1021/acsmeasuresciau.6c00075

**Published:** 2026-05-02

**Authors:** Khalid Haliru, Nicholas J. Goddard, Melissa Grant, Ana Poveda, Ruchi Gupta

**Affiliations:** † School of Chemistry, 1724 University of Birmingham, Birmingham B15 2TT, U.K.; ‡ Independent Researcher, Accrington BB5 2SB, U.K.; § School of Dentistry, University of Birmingham, Birmingham B15 2TT, U.K.

**Keywords:** hydrogel beads, quantitative, saliva, protein biomarkers, cytokines

## Abstract

We report a novel poly­(ethylene) glycol (PEG) hydrogel bead-based assay that permits measurement of protein biomarkers in complex samples such as saliva without sample processing and with limits of detection and dynamic range that are comparable or better than the current gold-standard, enzyme-linked immunosorbent assay (ELISA) without requiring detection antibodies and enzyme amplification. Additionally, in comparison to ELISA, the analysis time is >2 times shorter and the workflow is simpler. The PEG-based hydrogel beads preconcentrated protein biomarkers by covalent capture and in parallel removed interferent proteins by molecular weight cutoff without being affected by salts. The captured proteins were fluorescently labeled during their photochemical release from hydrogel beads. The released proteins were bound to immobilized antibodies and fluorescence was measured to determine their concentrations. Using the reported hydrogel beads, we measured proteins such as interleukin 6 (IL6) and interleukin 8 (IL8) at pg/mL concentrations in 200 μL of unprocessed saliva in ∼100 min. We applied the hydrogel beads to measure IL6 and IL8 in minimally stimulated saliva of oral lichen planus (OLP) patients and healthy individuals.

## Graphical abstract

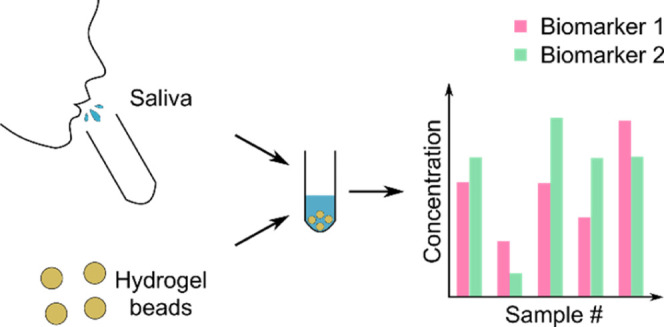

## 1 Introduction

Saliva is a promising alternate fluid to blood for disease diagnosis.^1,2^ Saliva collection is noninvasive, easy, and rapid.^3^ These advantages offered by saliva have increased its popularity for self-testing,^4^ mass screening,^5–7^ and for testing vulnerable individuals (e.g., children).^8,9^ Saliva contains salts, mucins, enzymes, and proteins.^10^ There is a growing scientific literature on the use of salivary biomarkers for detection of chronic diseases such as oral cancer.^11^ For example, the concentration of salivary cytokines such as interleukin 6 (IL6) and interleukin 8 (IL8) is reported to be higher in oral premalignant disorder (OPMD) and cancer patients than in healthy individuals.^12,13^

Protein biomarkers are commonly measured using enzyme-linked immunosorbent assay (ELISA),^14^ which offers limits of detection (LODs) in the range of pg/mL to ng/mL.^15^ ELISA, however, often suffers from nonspecific adsorption of interferents to microtiter plate wells^16^ and hence requires optimized washing and blocking conditions^17,18^ as well as sample processing (e.g., centrifugation, dilution, affinity capture to remove interferents).^19^ Furthermore, it is often essential to optimize protocols for each type of sample because of differences in interferences. For example, ELISA kits developed for measurements of biomarkers in cell culture supernatant and serum/plasma can result in intra- and inter-assay variations of >10% when used for saliva.^20^ Similarly, variations in saliva viscosity can result in over- or under-estimation of biomarker concentrations when measured using lateral and vertical flow immunoassays.^21,22^ To address this challenge, approaches such as the use of centrifugal pressure to transport sample^23^ and sample pretreatment^24^ have been reported but these are realized at the expense of increased complexity and instrumentation.

Equally, measurement of biomarkers using nanomaterials and/or hydrogels is gaining popularity.^25^ For example, colloidal gold nanoparticles that aggregate and hence change color in the presence of analytes have been reported.^26^ However, interferents in saliva can affect aggregation of colloidal nanoparticles.^26^ Hydrogels have been used as either high-loading immobilization matrices for recognition elements (e.g., antibodies)^27–31^ coupled to biosensors or as stimulus-responsive materials actively participating in the sensing process.^32,33^ Equally, hydrogels have been used for sample preparation as resins for removing highly abundant interferent proteins by affinity capture^34^ or molecular weight cutoff (MWCO).^35^ The authors have previously reported novel polyacrylamide and polyethylene glycol (PEG) hydrogels for preconcentration of protein analytes and removal of interferents simultaneously.^36,37^ The covalent capture of protein analytes was achieved via a reactive group attached to a fluorophore with the fluorophore attached to the hydrogel backbone by a light-cleavable bond (see Figure 1).^36^ As proteins were covalently captured from the sample, their concentration in the hydrogel increased over time until essentially all of the protein was captured by the hydrogel. Furthermore, as hydrogel volume was lower than sample volume, proteins were preconcentrated. The preconcentrated proteins were released by exposure to 365 nm light, breaking a bond between the fluorophore and a photoremovable group permanently tethered to the hydrogel backbone. Thus, as shown in Figure 1, the fluorophore remained attached to the protein, which implies that proteins released from the hydrogel were fluorescently labeled. Released proteins were measured by selective capture using antibody-coated microtiter plates and a fluorescence plate reader. In comparison to polyacrylamide, the PEG hydrogel offered a significantly improved LOD of sub ng/mL and multianalyte quantitation from a single sample.^37^ Furthermore, the MWCO of the PEG hydrogel was tailored to exclude albumin, immunoglobulins, and other high-molecular-weight interferents, allowing measurement of low-molecular-weight proteins in unprocessed serum.^37^

**Figure 1:**
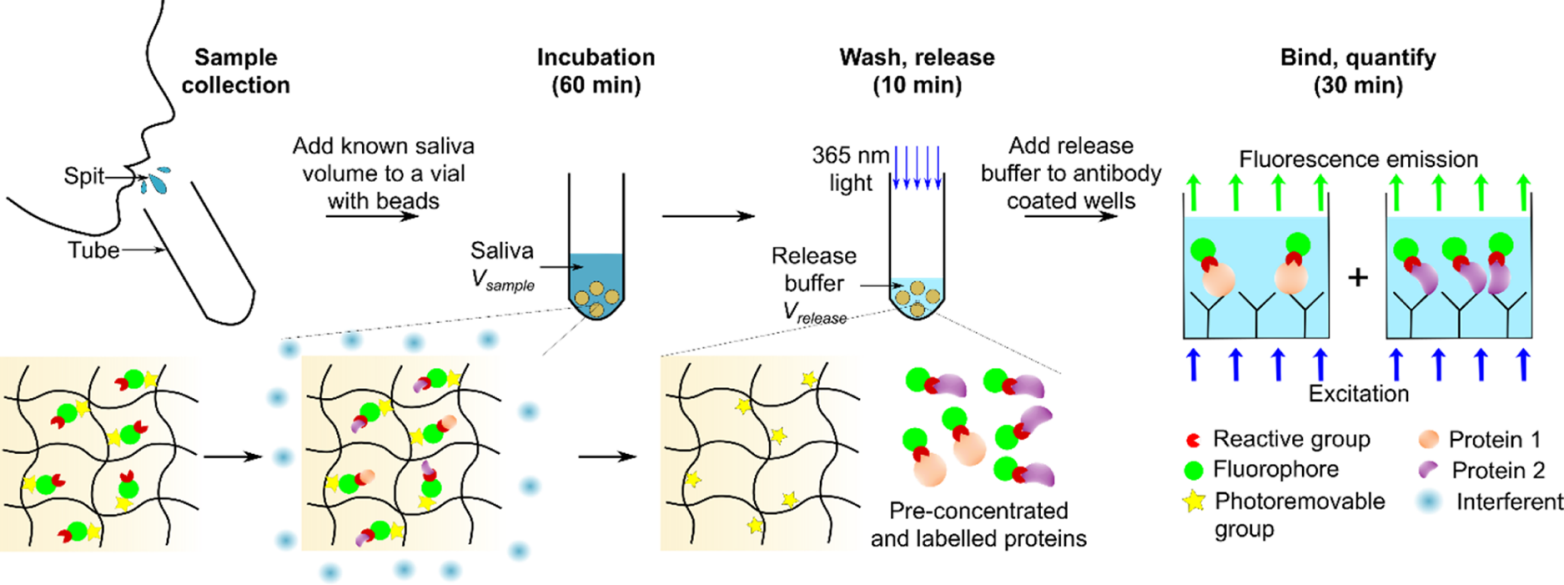
A schematic of the assay using PEG hydrogel beads for preconcentration and fluorescent labeling of proteins while excluding interferents in a body fluid, saliva, followed by release of proteins from hydrogels for selective capture by antibodies and quantitation by fluorescence (preconcentration factor, α *= V*_sample_/*V*_release_).

The work presented here is an unprecedented report of a PEG hydrogel with preconcentration, fluorescent labeling, and release functionality prepared in the form of beads for the measurement of salivary proteins. More specifically, the beads were shown to be suited for the measurement of IL6 and IL8 in unprocessed saliva of individuals suffering from a type of OPMD condition, oral lichen planus (OLP), and healthy controls. We used minimally stimulated saliva, which is more viscous and higher in proteins, metabolites, and microbiome content than stimulated saliva. Thus, minimally stimulated saliva presents significant analytical challenges, because of the sample complexity. A schematic showing the steps involved in the measurement of proteins using hydrogel beads is provided in Figure 1. The key steps were saliva collection, incubation of saliva with hydrogel beads for protein preconcentration and removal of interferents, release of captured proteins that are fluorescently labeled, capture of selected released proteins by antibodies, and fluorescence measurement for quantitation.

The hydrogel beads reported in this work offered LOD at pg/mL levels, which is about 2 orders of magnitude better than our previous work.^37^ Additionally, the sample volume used in this work was 200 μL compared to 10 mL in our previous work.^37^ These are significant improvements because the physiological concentrations of many protein biomarkers including IL6 and IL8 are at the pg/mL level and 200 μL minimally stimulated saliva samples can be collected in a few minutes^38^ simply by spitting in tubes. We showed that an incubation time of 60 min is sufficient to capture >95% proteins in hydrogel beads while removing interferents in saliva. Equally, we showed that proteins can be released from hydrogel beads in 10 min by exposure to 365 nm light. Released proteins are fluorescently labeled, and their concentrations can be determined by binding to immobilized primary antibodies and measuring fluorescence without requiring enzyme-labeled secondary antibodies, enzyme substrate, and stop solution as is the case for ELISA. The overall time for protein measurement using hydrogel beads was ∼100 min, which is >2 times faster than common commercially available ELISA kits. Furthermore, the measurement of salivary proteins using hydrogel beads did not require sample processing and did not suffer from interferents in saliva. Finally, hydrogel beads can be stored dry for at least up to 6 months and proteins captured in hydrogel beads are significantly more stable than in saliva.

## 2 Experimental Section

### 2.1 Materials

4-Arm poly­(ethylene glycol)-dibenzocyclooctyne (PEG-DBCO) (PSB-4071, molecular weight: 5000 g/mol) was bought from Creative PEGWorks. PEG bis-azide and FITC-NVOC-PEG-azide (molecular weight: ∼4105 g/mol) inactive and active monomers, respectively, were synthesized in-house using the procedure reported previously.^37^ More specifically, PEG bis-azide of molecular weights 2050, 6050, 10 050, and 20 050 g/mol were synthesized. For the FITC-NVOC-PEG-azide monomer, after extraction with DCM, the monomer was dialyzed using SnakeSkin tubing with 3500 Da MWCO (68035, Thermo Fisher Scientific) for 3 days with deionized water changed every 6 h and then lyophilized. Phosphate buffered saline (PBS, J62036.K3) was purchased from Fisher Scientific. Ethanolamine, PEG with a molecular weight of 300 000 g/mol, and trehalose were bought from Merck. ELISA kits for IL6 (SimpleStep ab178013, ab46027, and high-sensitivity ab46042) were bought from Abcam. ELISA kit for IL8 (DY208) and ancillary reagent kit (DY008B) were bought from Bio-Techne.

Saliva was collected from healthy volunteers and OLP patients visiting the University of Birmingham Dental Hospital (UK) with approval from the local research ethics committee (ethics code 24/SW/0100, project code DRTB002.2029). The healthy volunteers and OLP patients were in age groups 30–45 and 30–81 years, respectively. The male:female ratios of healthy volunteers and the ratios of the OLP patients were 40:60 and 25:75, respectively. Individuals were asked to spit into a sterile container for ∼10 min to collect at least 1 mL of saliva. Samples were placed on ice immediately after collection and aliquoted into 500 μL volumes. The aliquots were stored in an −80 °C freezer prior to use.

### 2.2 Fabrication of PEG Hydrogel Beads

Hydrogel precursor solution was prepared by dissolving 4-arm PEG DBCO, PEG bis-azide, and FITC-NVOC-PEG-azide in deionized water. The quantities used for making 1 mL of 5% (w:v) precursor solution are summarized in Table 1. The molar ratio of PEG bis-azide to 4-arm PEG DBCO was ∼2:1. The molar ratio of the active monomer to total monomers and cross-linker was 1:40. The solution was vortexed and used immediately. Each bead was made by pipetting a 4 μL drop of the hydrogel precursor solution on a hydrophobic surface. The drops were left on the surface for ∼30 min in the dark to allow polymerization. The resulting beads were stored dry in the dark until use.

**Table 1:** Composition of 1 mL of 5% (w:v) Hydrogel Precursor Solution

Chemical	Molecular weight of the inactive monomer (g/mol)			
	2050	6050	10 050	20 050
4-arm PEG DBCO (cross-linker)	27.4 mg	14.9 mg	10.2 mg	5.7 mg
PEG bis-azide (inactive monomer)	21.1 mg	34.3 mg	39.2 mg	44.0 mg
FITC-NVOC-PEG-azide (active monomer)	1.5 mg	0.8 mg	0.6 mg	0.3 mg
H_2_O	1.0 mL	1.0 mL	1.0 mL	1.0 mL

### 2.3 Procedure

Dried hydrogel beads were incubated in protein solutions while being agitated using a nutating shaker (BCM1610, Generon). Unless stated otherwise, 4 hydrogel beads made using 5% (w:v) precursor solution were used, the protein solution was IL6 dissolved in PBS buffer, IL6 concentration was 50 pg/mL, and sample volume was 200 μL. After incubation, hydrogel beads were washed in 200 μL of PBS for 5 min, immersed in 50 μL of PBS, and exposed to 365 nm light (Relybo 30 W rechargeable UV torch, Amazon) for 10 min to release proteins from hydrogel beads to buffer. The beads were transferred between solutions manually. The release buffer containing proteins was pipetted in wells coated with capture antibody available as part of the ELISA kits. After 30 min, the release buffer was removed, and wells were washed three times with 200 μL of PBS buffer. Fluorescence spectra of wells were then measured using a CLARIOstar Plus microplate reader (BMG Labtech) with the excitation wavelength set to 490 nm. The same procedure was used to measure IL8 using hydrogel beads. An anti-IL8-coated microtiter plate was prepared by pipetting 100 μL of 4 μg/mL antibody solution in PBS. The plate was sealed and incubated overnight. Subsequently, the manufacturer’s protocol was followed to wash and block the plate before use.

To perform IL6 and IL8 ELISA, manufacturer’s protocols were followed. This involved incubating analyte solution to antibody-coated microtiter plates, detection antibody labeled with biotin, and streptavidin labeled with horseradish peroxidase with intermediate washing. Subsequently, 3,3′,5,5′-tetramethylbenzidine substrate solution was added to wells and incubated for 20 min, and sulfuric acid stop solution was added. Buffer washes were performed, and finally, absorbance of each well was measured at 450 nm. The typical time for ELISA was ∼225 min, with incubation with detection antibody being the longest.

Viscosity of solutions was measured using an A&D Weighing SV-100 viscometer, absorption of protein solutions at 280 nm was measured using a Nanodrop 1000 spectrophotometer, and the absorbance of solutions in microtiter plates was measured using a spectrometer (BioTek ELx800 microplate reader). To shake solutions in microtiter plates, a vortex mixer (S0200-230 V-UK, Labnet) with an optional head for holding a microtiter plate was used. Hydrogel beads were imaged using a Motic BA310 LED Microscope and images were analyzed using ImageJ 1.54p. Graphs were plotted using either SigmaPlot 10.0 or OriginPro 2024, and illustrations were created using Inkscape 1.4.

## 3 Results and Discussion

### 3.1 Characterization of Hydrogel Beads

The hydrogel beads were formed by strain-promoted azide–alkyne click chemistry (SPAAC) reaction between 4-arm PEG DBCO cross-linker and monomers. The chemical structures of the cross-linker and monomers are shown in Figure 2a. PEG bis-azide and FITC-NVOC-PEG-azide were used as inactive and active monomers, respectively. In comparison to our previous work, the use of 4-arm PEG DBCO as a cross-linker allowed the hydrogel to be formed by a copper-free click reaction.^37^ Thus, hydrogel beads reported in this work are free of cytotoxic transition metal, making them attractive for applications such as in vivo capture of protein biomarkers, which may be explored in the future. In this work, hydrogel beads were used for the capture of proteins in in vitro samples. Proteins were covalently captured in hydrogel beads by reaction between primary amines in proteins and isothiocyanate on FITC in the active monomer incorporated in hydrogel beads. The reaction between isothiocyanate and amine does not produce any byproducts.^39^ Proteins were released from hydrogel beads by photolytic cleavage of a carbonate bond between FITC and NVOC, releasing proteins labeled with fluorescein as shown in Figure 2b. In comparison to carbamate, which is another commonly used photochemically cleavable bond, carbonate is beneficial because addition of toxic and carcinogenic semicarbazide in the release buffer is not required.^40^

**Figure 2:**
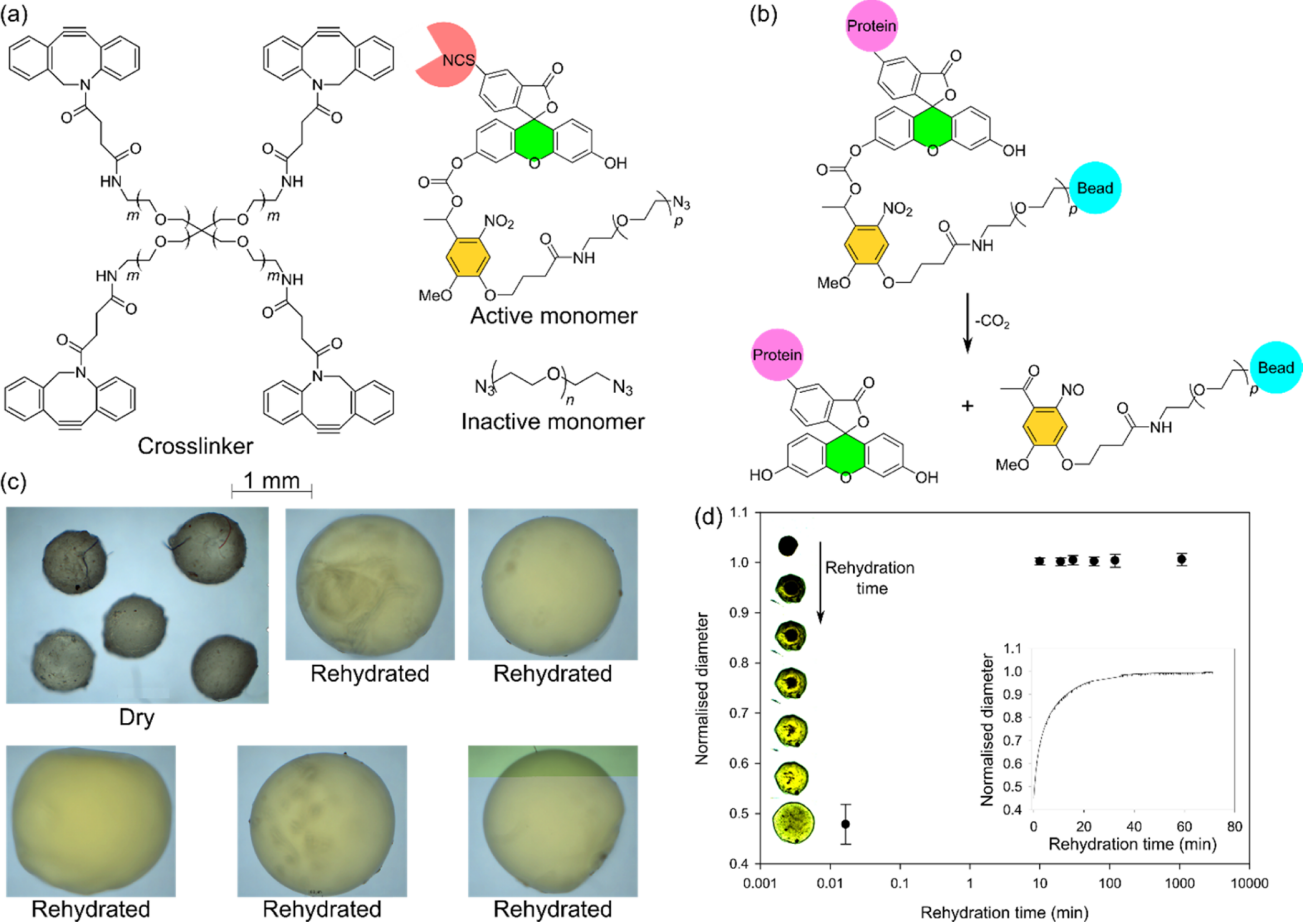
(a) Chemical structures of the cross-linker and monomers used to form hydrogel beads (*m* = 21, *n* = 45 or 136 or 227 or 454, and *p* = 76), (b) reaction scheme showing photolytic release of fluorescein-labeled proteins from beads, (c) microscope images of dried and 24 h rehydrated beads, and (d) radius of hydrogel beads versus rehydration time in PBS (error bars were calculated using 10 beads and the inset shows the rehydration curve of a bead where images were saved every second over a total duration of 71.3 min).

Typically, 0.5 mL of hydrogel precursor solution was prepared at any one time. The SPAAC reaction was sufficiently slow to dispense all of the 0.5 mL hydrogel precursor solution as 4 μL drops on a hydrophobic surface to make beads. Thus, ∼125 beads were formed at any one time. We tried to make beads by pipetting ≤3 μL precursor solution, but it was challenging to dislodge such small volumes from the end of a pipet tip to a hydrophobic surface. Thus, beads fabricated by dispensing 4 μL of precursor solution were used for the remainder of this work. After polymerization, beads were stored dry in darkness until use. Figure 2c shows microscope images of dried beads and of the same beads after rehydration in PBS for 24 h. Images were analyzed using the particle finder functionality of ImageJ after conversion to greyscale and thresholding. The radii of dried and 24 h rehydrated beads were 491 ± 27 and 1029 ± 46 μm, respectively. Thus, variations in the radius of dried and rehydrated beads were ∼5.5% and ∼4.4%, respectively. Significantly smaller radius beads can be manufactured in large numbers using droplet microfluidics^41^ and will be investigated in future studies. Assuming that the beads were spherical, volumes of dried and 24 h rehydrated beads were 0.5 ± 0.1 and 4.6 ± 0.6 μL, respectively. The volume of the rehydrated beads is comparable to the original volume of the precursor solution used to make them, suggesting that the beads were fully swollen by overnight rehydration.

Next, we studied the swelling kinetics of the hydrogel beads. For this purpose, we made 10 beads, dried them overnight, and took microscope images. Afterward, we rehydrated the beads by immersing in PBS and took images between 10 and 1080 min. The images were analyzed using ImageJ to find their area and hence diameter. A plot of normalized diameter versus rehydration time of the beads is provided in Figure 2d, suggesting that the beads were largely rehydrated in ∼10 min. To study the swelling kinetics in more detail, we selected one of the dry beads and rehydrated it in PBS while recording images every second for a total duration of 71.3 min. The images were analyzed using ImageJ to extract diameter, and a plot of normalized diameter as a function of rehydration time for the beads is provided in the inset in Figure 2d. The data was fitted to 5-parameter double exponential. Double exponential was selected because, as shown by the beads images, the interior of the beads swelled at a slower rate than the surface. The first and second time constants were determined to be 1.7 and 10.6 min, respectively.

### 3.2 Assay Development

Protein measurement using hydrogel beads is a total quantitation assay; i.e., the assay is suited for determining the amount of a protein in a sample or protein concentration in known sample volume. The moles of the active monomer in each hydrogel bead were much greater than the moles of proteins in each sample. This implies that the protein concentration in a sample (*c*_sample_) can be determined using eq 1.

1 $$c_{sample}=c_{release}\frac{V_{release}}{V_{sample}}$$

where *c*_release_ is the concentration of the protein in the release buffer and *V*_sample_ and *V*_release_ are the volumes of sample and release buffer, respectively. As proteins released from hydrogel beads are fluorescently labeled, *c*_release_ is determined by allowing the released protein to bind to antibodies and subsequently measuring the fluorescence intensity. Equation 1 highlights that *c*_release_ can be higher than *c*_sample_ if *V*_release_ < *V*_sample_. This implies that the preconcentration factor (α) is given by eq 2.

2 $$\alpha =\frac{V_{sample}}{V_{release}}$$

In this work, *V*_sample_ and *V*_release_ were 200 and 50 μL, respectively. Hence, the α value was 4. The key performance parameters for protein measurement using hydrogel beads are assay time, detection sensitivity, LOD, and ability to exclude the effects of interferents. These are discussed below.

#### 3.2.1 Assay Time

The assay time is determined by incubation, release, and binding times. Each of these times were determined using 50 pg/mL IL6 solutions prepared in PBS buffer as discussed below.

- Incubation time: The incubation time is the time for which dried hydrogel beads should be immersed in sample solutions to capture IL6. If incubation time is low, less IL6 will be captured and hence the concentration of the protein in the release buffer will also be low. Additionally, as IL6 released from hydrogel beads was labeled with fluorescein, the lower the concentration of IL6 in the release buffer, the lower will be the fluorescence intensity. As expected, the fluorescence intensity of IL6 released from hydrogel beads increased as the incubation time was increased from 10 to 60 min (see Figure 3a). Subsequently, there was an insignificant increase in the fluorescence intensity with the incubation time because all the IL6 was captured within 60 min. A plot of peak fluorescence intensity versus incubation time (inset in Figure 3a) was fitted to an exponential rise to a maximum, which suggested that 95% of the IL6 was captured by hydrogel beads in 52.5 min. Thus, an incubation time of 60 min was used to ensure >95% capture of proteins by hydrogel beads.
- Release time: The release time is determined by the choice of the photoremovable protecting group and photocleavable bond, which are a nitroveratryl derivative and carbonate, respectively (see Figure 2b), as well as the power density of a 365 nm light source. Compared to our previous work, we used a higher power density light source (∼2100 mW/cm^2^ in this work versus ∼210 mW/cm^2^ in the previous work).^36,37^ As shown in Figure S1 of the Supporting Information, 90% of the protein was released in ∼7.2 min compared to ∼23.4 min in our previous work.^37^ Thus, to ensure that >90% of proteins are released from hydrogel beads, the release time was selected to be 10 min.
- Binding time: To compare the performance of hydrogel beads directly against ELISA, we used antibody-coated microtiter plates supplied with IL6 ELISA kits to capture IL6 released from hydrogel beads. IL6 in release buffer was allowed to bind to anti-IL6-coated wells for 30 min and then fluorescence was measured to quantify IL6.

**Figure 3:**
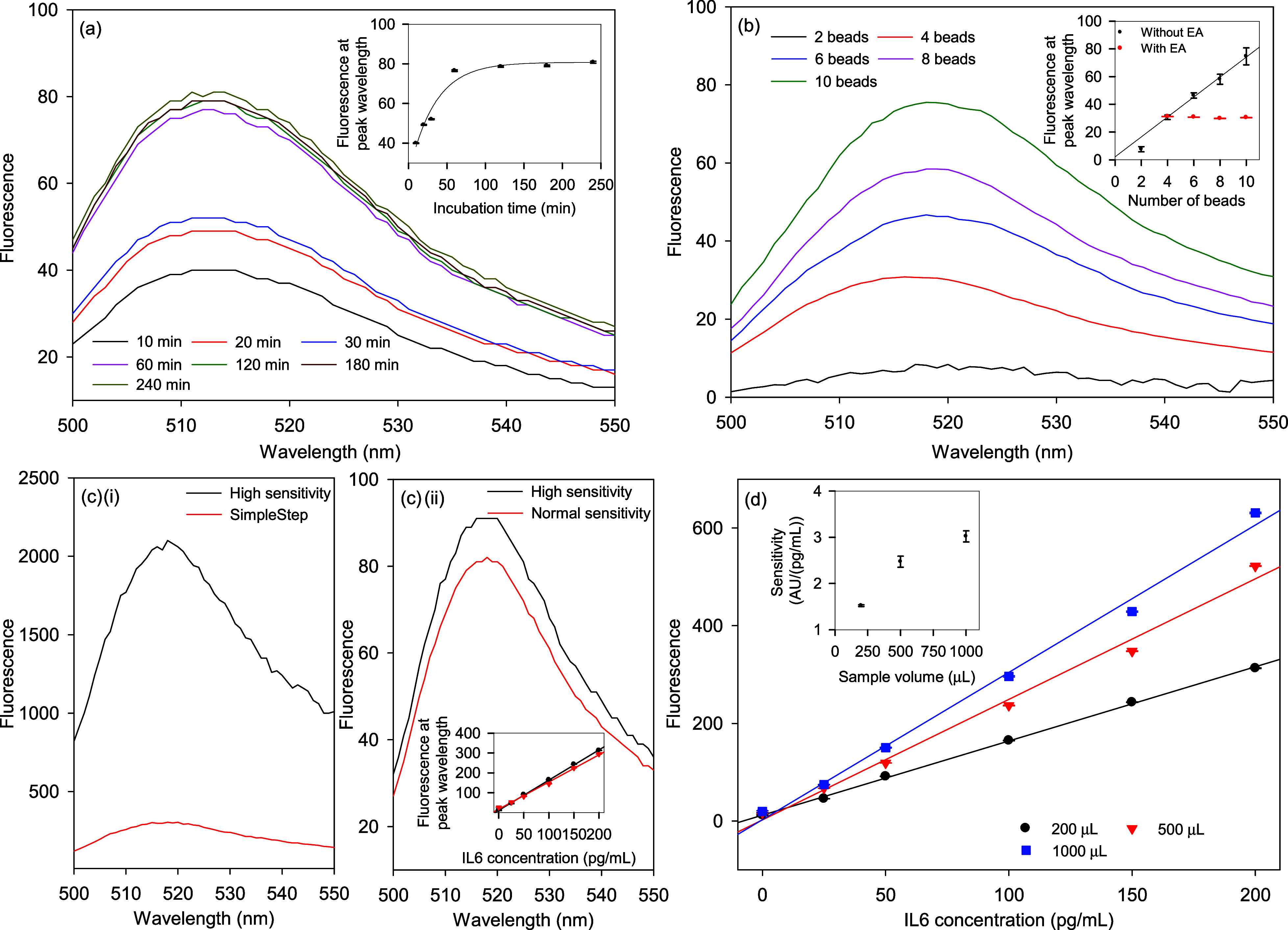
Fluorescence spectra of IL6 solutions released from hydrogel beads for different (a) incubation times, (b) number of beads, (c) antibody-coated microtiter plates available as part of three ELISA kits where in concentration of IL6 was (i) 5 ng/mL and (ii) 50 pg/mL, and (d) calibration curves of IL6 for different solution volumes.

Thus, the overall assay time for measurement of proteins using hydrogel beads was ∼100 min that included 60 min of incubation time, 10 min of release time, and 30 min of binding time. The overall assay time using hydrogel beads is significantly lower than high sensitivity ELISA (e.g., ab46042 used in this work) that takes 225 min while offering a comparable LOD (discussed below). Similarly, the assay time for IL8 ELISA was 280 min.

#### 3.2.2 Detection Sensitivity and LOD

The detection sensitivity and LOD are affected by factors such as number of beads, choice of antibodies, and sample volume (or preconcentration factor, α) as shown in Figure 3b–d, respectively. The effect of each of these factors is discussed below.

- Number of beads: Figure 3b shows that as the number of beads was increased from 2 to 10, fluorescence intensity of released IL6 increased, which was unexpected. This increase in the fluorescence signal cannot be attributed to the higher capture of IL6. This is because, for 50 pg/mL IL6 solution of 200 μL, the number of moles of IL6 is 480 attomoles, which is much less than the number of moles of isothiocyanate (1.5 nanomoles) of the active monomer in each bead and hence a single bead would have been sufficient to capture all the IL6 in the sample solution. To explain our observation of an increase in fluorescence signal with the number of beads, we hypothesized that the increase in fluorescence with the number of beads was a result of an increase in the degree of labeling. When hydrogel beads are exposed to 365 nm light, unreacted FITC was released along with IL6 and can react with amines in the protein. To prevent this and to “mop up” unreacted FITC, beads can be washed with an amine-containing buffer before proteins are released. A comparison of fluorescence for 4 to 10 beads for when beads were immersed in PBS without and with 0.1% ethanolamine after protein capture, but before release, is shown in Figures S2 and 4.
Figure 4 shows that fluorescence was independent of the number of beads when beads were immersed in 0.1% ethanolamine before IL6 was released. However, fluorescence increased linearly with the number of beads when ethanolamine was absent in the buffer, confirming that the increase in fluorescence signal was because of the increase in the degree of fluorescent labeling. Equally, Figure 4 suggests that the degree of labeling for IL6 can be controlled by changing the number of hydrogel beads. It was not possible to use more than 10 beads because this was the maximum number of beads that could be fully submerged in 50 μL of release buffer. As the fluorescence in the absence and presence of ethanolamine was the same when 4 beads were used, the remainder of the work was carried out using 4 beads. Using a greater number of beads to increase the degree of labeling may affect the binding of a protein to an antibody. While increasing the degree can increase the detection sensitivity, it may not lead to improved LOD.

**Figure 4:**
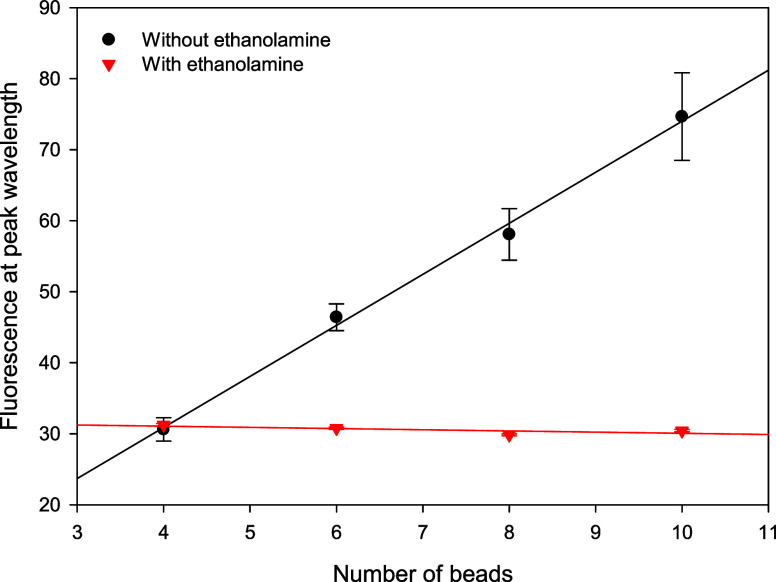
Fluorescence at peak wavelength of released IL6 versus number of beads when beads were immersed in PBS without and with 0.1% ethanolamine before protein release.

- Choice of antibodies: We used anti-IL6 provided in commercial ELISA kits for selective capture and quantification of IL6 released from hydrogel beads. We used three types of IL6 ELISA—SimpleStep, normal sensitivity, and high sensitivity kits. As shown in Figure 3c­(i), fluorescence intensity of IL6 released from hydrogel and after binding to antibodies available in SimpleStep was significantly lower than those in a high-sensitivity ELISA kit, suggesting that antibodies in SimpleStep had lower affinity for IL6 than the high sensitivity kit. As a result, the antibodies supplied in the SimpleStep when combined with hydrogel beads were only suited to measure IL6 in the ng/mL range. A comparison of the fluorescence spectra of released IL6 bound to antibodies in high and normal sensitivity ELISA kits suggested a small difference in their binding affinities (see Figure 3c­(ii)). The inset in Figure 3c­(ii) provides calibration curves of IL6 released from hydrogel beads and bound to antibodies in high and normal sensitivity ELISA kits. A summary of the calibration curves is provided in Table 2. A *t* test suggested that detection sensitivity obtained using antibodies supplied in high and normal sensitivity ELISA kits was significantly different at the 95% confidence level. Furthermore, as highlighted in Table 2, the LOD obtained using antibodies supplied in high sensitivity kit was ∼2.3 better than the normal sensitivity kit.

**Table 2:** Calibration Curves of IL6 Released from 5% (w:v) Hydrogel Beads Where the Released Protein Was Bound to Antibody-Coated Microtiter Plates Supplied in Different ELISA Kits, *F* is the Fluorescence Intensity in Arbitrary Units (AU) and *c* Is the Protein Concentration in pg/mL

Antibodies available	Best fit line (coefficient of determination)	Detection sensitivity (AU/(pg/mL))	LOD (pg/mL)
High-sensitivity ELISA kit	*F* = 11.96 + 1.52×*c* (0.9993)	1.52 ± 0.02	4.6
Normal sensitivity ELISA kit	*F* = 18.5 + 1.36×*c* (0.9961)	1.36 ± 0.04	10.5

In summary, antibodies available in SimpleStep were not suited for the measurement of IL6 at physiological levels because they resulted in a LOD of ng/mL. While the LOD obtained using antibodies in the high sensitivity kit was better than the normal sensitivity kit, the latter was used for the remainder of this work because it is more readily available.

- Sample volume: As the volume of IL6 solution increases, the amount of the protein in the solution increases. Thus, the amount of IL6 captured by hydrogel beads should increase with sample volume. This in turn implies that the concentration of IL6 in the release buffer and detection sensitivity is expected to increase linearly with the sample volume. To study this, we prepared IL6 solutions of different concentrations and incubated 4 beads in each concentration of IL6 solution of different volumes for 60 min with agitation. In each case, IL6 was released into 50 μL of buffer, captured using anti-IL6-coated microtiter plates, and then fluorescence spectra were measured. Fluorescence spectra of released IL6 solutions for different sample volumes are shown in Figures S3–S5 in the Supporting Information. The fluorescence at peak wavelength versus IL6 concentration was then plotted for different solution volumes with the resulting graphs shown in Figure 3d.
  A summary of the calibration curves for each sample volume is provided in Table 3. Table 3 and the inset in Figure 3d highlight that, as expected, the detection sensitivity increased with sample volume. However, the relationship between the detection sensitivity and sample volume was not linear and appeared to be reaching a saturation value. This may be because the concentration of IL6 in the release buffer was high enough to begin to saturate the antibodies. Furthermore, although the detection sensitivity was lower for the 200 μL sample volume, the LOD was better than 500 and 1000 μL (see Table 3). Thus, the remainder of the work was performed using 200 μL sample volume.

**Table 3:** Calibration Curves of IL6 Released from Hydrogel Beads Incubated with Different Sample Volumes and Antibodies Available in the High Sensitivity ELISA Kit Were Used, *F* is the Fluorescence Intensity in Arbitrary Units (AU) and *c* Is the Protein Concentration in pg/mL

Sample volume (μL)	Best fit line (coefficient of determination)	Detection sensitivity (AU/(pg/mL))	LOD (pg/mL)
200	*F* = 11.96 + 1.52×*c* (0.9993)	1.52 ± 0.02	4.6
500	*F* = 1.95 + 2.47×*c* (0.9906)	2.47 ± 0.12	16.4
1000	*F* = 2.74 + 3.02×*c* (0.9938)	3.02 ± 0.12	13.3

#### 3.2.3 Alleviating the Effect of Interferents

Measurement of proteins in saliva can be negatively impacted by physical and chemical interferents. For example, protein measurement can be affected by saliva viscosity, which can vary between 1.5 mPa·s and 23 mPa·s,^42^ and is largely determined by the concentration of macromolecular glycoproteins such as mucins. The key chemical interferents in saliva include salts, enzymes, and proteins other than biomarkers. We have previously shown that the measurement of proteins using the PEG hydrogel is unaffected by salts.^37^ Below, we discuss solutions for alleviating the effects of viscosity and protein interferents on protein measurement using hydrogel beads.

- Physical—viscosity: To study the effect of sample viscosity, we prepared 50 pg/mL IL6 in PBS containing different concentrations of a viscosity-enhancing substance, 300 000 g/mol PEG, which mimicked macromolecular glycoproteins in saliva. The viscosity of different concentrations of PEG solutions is summarized in Table S1 in the Supporting Information. Fluorescence spectra of IL6 prepared in different concentrations of PEG solutions are shown in Figure S6 in the Supporting Information. The corresponding peak fluorescence intensity of IL6 as a function of viscosity of PEG solutions is plotted in Figure 5a. Figure 5a suggests that when IL6 was captured from viscous solutions, the protein concentration was underestimated. We hypothesized that this under-estimation is because the higher solution viscosity slows the movement of the beads and diffusion of proteins (in this case, IL6) in solutions. Our hypothesis was confirmed by the data presented in Figure 5b, which shows that the peak fluorescence of IL6 captured from viscous solution increased when hydrogel beads were incubated with 23 mPa·s IL6 solution for 18 h than for 2 h.
- Chemical—interferent proteins: Saliva contains proteins such as mucins and enzymes (e.g., amylase), which are present in large excess (1.2 mg/mL^43^ and 0.04–0.4 mg/mL,^44^ respectively). These proteins can interfere with the measurement of protein biomarkers such as cytokines. As cytokines are low molecular weight while interferent proteins are high molecular weight, one way to remove interferent proteins is by MWCO. For example, the molecular weights of IL6 and IL8 are ∼20 000 g/mol and ∼8900 g/mol, respectively, while those of mucins and amylase are 10^6^ g/mol and ∼60 000 g/mol, respectively. The hydrogel can be designed to exclude high-molecular-weight proteins and in parallel capture proteins that can diffuse in beads. For this purpose, we studied the effect of total weight to volume percentage of hydrogel and molecular weight of PEG bis-azide on percentage recovery of proteins of different molecular weights. The percentage exclusion is (100—percentage recovery of proteins). We previously studied the effect of total weight to volume percentage of hydrogel on percentage recovery of different molecular weight proteins, but the hydrogel was in the form of discs,^37^ and the effect of the molecular weight of PEG bis-azide was not studied. It is important to characterize percentage recovery of proteins for beads because their surface to volume ratio is higher than that of discs, 2.9 mm^–1^ per bead and 1.5 mm^–1^ per disc. This implies that proteins that cannot diffuse in beads can still be captured on the surface of beads, which in turn can affect their percentage recovery and hence exclusion.
  We studied the percentage recovery of proteins of different molecular weights from the hydrogel beads. For this purpose, 0.1 mg/mL solution of each protein was prepared in PBS and its absorption at 280 nm was measured. Four beads made of selected total weight to volume percentage hydrogel were incubated with 200 μL of each protein solution for 60 min while being agitated. After incubation, beads were washed in PBS for 5 min, immersed in 200 μL of PBS, and exposed to 365 nm light for 10 min to release the proteins. The absorption at 280 nm of the release buffer containing each protein was measured. The ratio of absorbances of the release buffer and the original protein solution provided the percentage protein recovery, which are tabulated in Tables S2–S5 in the Supporting Information.
  A plot of percentage protein recovery versus molecular weight of proteins for different total weight to volume of hydrogel beads containing 2050 g/mol PEG bis-azide is shown in Figure 6a. Figure 6a shows that for 5% (w:v) and 10% (w:v) hydrogel beads, the percentage recovery of proteins was independent of the molecular weight and was >90%. This contrasts with hydrogel discs where the recovery of proteins of molecular weights >60 000 g/mol was significantly reduced.^37^ The data for 20% (w:v), 30% (w:v), and 40% (w:v) hydrogel beads was fitted to two-parameter exponential decay to determine the MWCO. MWCO was defined as the molecular weight of protein at which percentage recovery was dropped to ∼63% of the maximum value.
  The same trends were observed for beads containing other molecular weight PEG bis-azide (i.e., the inactive monomer in hydrogel beads). A plot of MWCO versus total weight to volume of hydrogel beads and molecular weight of PEG bis-azide is shown in Figure 6b. Figure 6b highlights that the total weight to volume of hydrogel has a stronger effect than the molecular weight of PEG bis-azide on the MWCO. Furthermore, the effect of the molecular weight of PEG bis-azide on the MWCO was low for high total weight to volume of hydrogel beads. For example, for 20% (w:v) and 40% (w:v) hydrogel beads, the MWCO changed by ∼40% and ∼15%, respectively, as the molecular weight of PEG bis-azide was varied from 2050 to 20 050 g/mol in both cases. Thus, the total weight to volume of hydrogel beads and the molecular weight of PEG bis-azide can be tuned to exclude interferents of molecular weight greater than analytes. For example, the MWCO of 20% (w:v) hydrogel beads containing 2050 g/mol PEG bis-azide was ∼24 000 g/mol. This implies that these hydrogel beads will be permeable to IL6 and IL8 but exclude mucin and amylase. Thus, the measurement of salivary IL6 and IL8 was carried out using 20% (w:v) beads containing 2050 g/mol PEG bis-azide.

**Figure 5:**
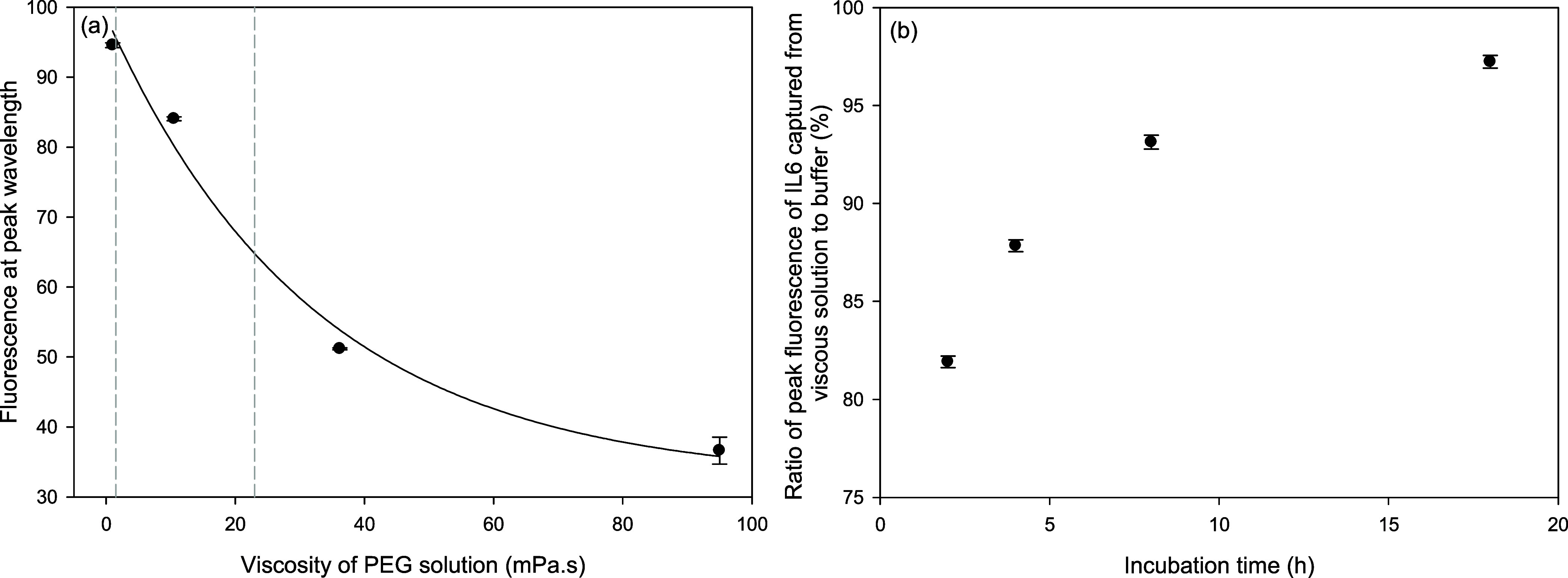
(a) Peak fluorescence intensity of IL6 released from beads that were incubated with 50 pg/mL IL6 prepared in different viscosity PEG solutions (gray lines indicate the range of viscosity values reported for saliva) and (b) plot of the ratio of peak fluorescence of IL6 captured from 23 mPa·s viscous solution to buffer versus incubation time.

**Figure 6:**
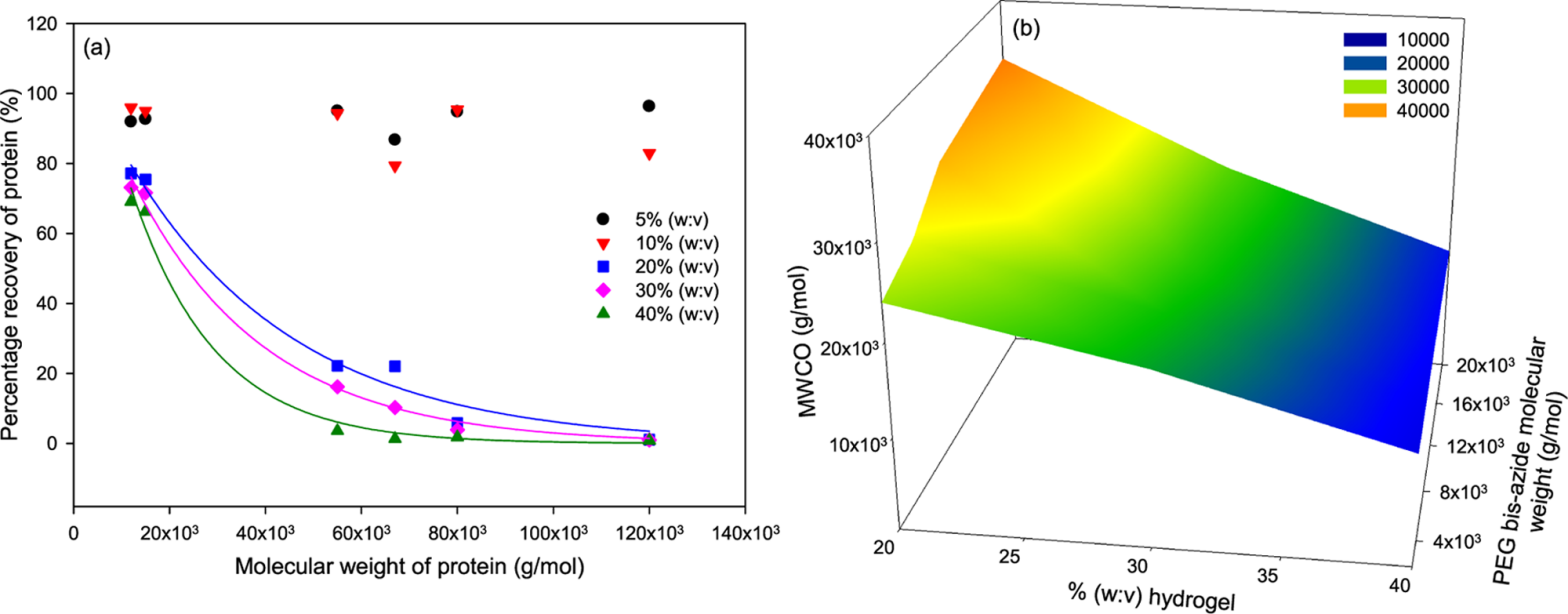
Plots of (a) percentage recovery of different molecular weight proteins for different total weight to volume of beads containing 2050 g/mol PEG bis-azide and (b) MWCO versus total weight to volume of hydrogel beads versus molecular weight of PEG bis-azide.

### 3.3 Measurement of Cytokines in Saliva

We measured the concentration of two cytokines, IL6 and IL8, in saliva of OLP individuals and healthy controls using 20% (w:v) hydrogel beads containing 2050 g/mol PEG *bis*-azide.

First, calibration curves of both proteins when measured using hydrogel beads and ELISA were determined. The calibration curves are shown in Figures S7–S10 in the Supporting Information and summarized in Table 4. Table 4 highlights that the LOD of IL6 using hydrogel was 10.1 pg/mL, which is two times better than ELISA. For IL8, the hydrogel beads offered wider dynamic range but higher LOD than ELISA. ELISA required a 50 μL sample, but a 200 μL sample volume was used for hydrogel beads.

**Table 4:** A Summary of the Calibration Curves of IL6 and IL8 Prepared in PBS for Hydrogel Beads and ELISA (*A* and *F* Are the Absorbance Fluorescence Intensity, Respectively, in AU and *c* Is the Concentration in pg/mL)

Protein	Method	Best fit line (coefficient of determination)	Sensitivity (AU/(pg/mL))	LOD (pg/mL)	Sample volume (μL)	Dynamic range (pg/mL)
IL6	Hydrogel beads	*F* = 15.5 + 1.02×*c* (0.9984)	1.02 ± 0.02	10.1	200	0–500
	ELISA	*A* = 0.09 + 5.53 × 10^–3^×*c* (0.9928)	5.53 × 10^–3^ ± 0.20 × 10^–3^	21.4	50	0–500
IL8	Hydrogel beads	*F* = 15.0 + 0.94×*c* (0.9912)	0.94 ± 0.04	33.3	200	0–500
	ELISA	*A* = 0.04 + 6.80 × 10^–3^×*c* (0.9954)	6.80 × 10^–3^ ± 0.30 × 10^–3^	15.3	50	0–250

The concentrations of salivary IL6 and IL8 measured using hydrogel beads for the OLP and healthy individuals are shown in Figure 7.

**Figure 7:**
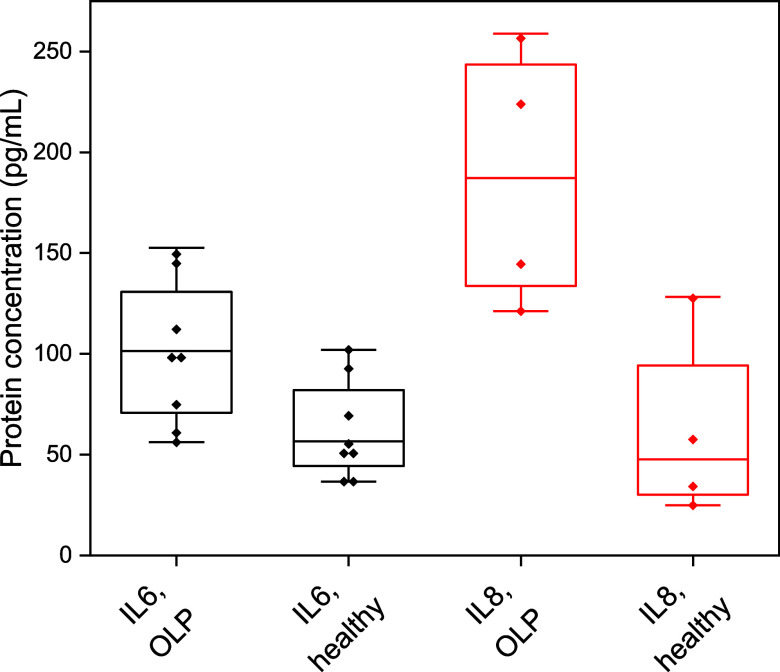
IL6 and IL8 concentrations in saliva samples of OLP and healthy individuals determined using hydrogel beads (unprocessed saliva samples were used).

Unprocessed saliva samples were used; i.e., the samples were neither diluted nor centrifuged nor treated in any other way. As shown in Figure 7, the median concentrations of IL6 in saliva of OLP and healthy individuals were 101.5 pg/mL and 56.6 pg/mL, respectively. Similarly, the median concentrations of IL8 in saliva of the patients with OLP and healthy individuals determined using hydrogel beads were 187.3 and 62.1 pg/mL, respectively. Higher IL6 and IL8 concentrations in saliva of OLP individuals than healthy controls are in line with the literature.^45,46^ These results should be read with caution, because the number of samples used in this study was small. The aim of this work, however, is to demonstrate that hydrogel beads can measure salivary proteins and not to discover/validate salivary biomarkers of OLP.

Next, we compared salivary IL6 and IL8 concentrations determined using hydrogel beads against the values determined using the gold-standard method, ELISA. Figure 8a is a plot of IL6 concentrations determined using hydrogel beads versus ELISA, which highlights that there are two saliva samples where the IL6 concentration appeared to be significantly different than the expected range when measured using ELISA but not hydrogel beads (marked by arrows in Figure 8a). This implies that the hydrogel beads reported in this work did not suffer from interferents in saliva, but in some cases, ELISA did. The two cases for which ELISA suffered from interferents were excluded to obtain Figure 8b and the slope of the best fit line was 0.91 ± 0.08 (*n* = 14 samples). A similar plot of salivary IL8 concentrations determined using hydrogel beads and ELISA is provided in Figure 8c. In this case, the slope of the best fit line was 1.06 ± 0.05 (*n* = 8 samples). Based on a *t* test, there was a significant difference in the concentrations of salivary IL6 and IL8 determined using hydrogel beads and ELISA at 95% confidence.

**Figure 8:**
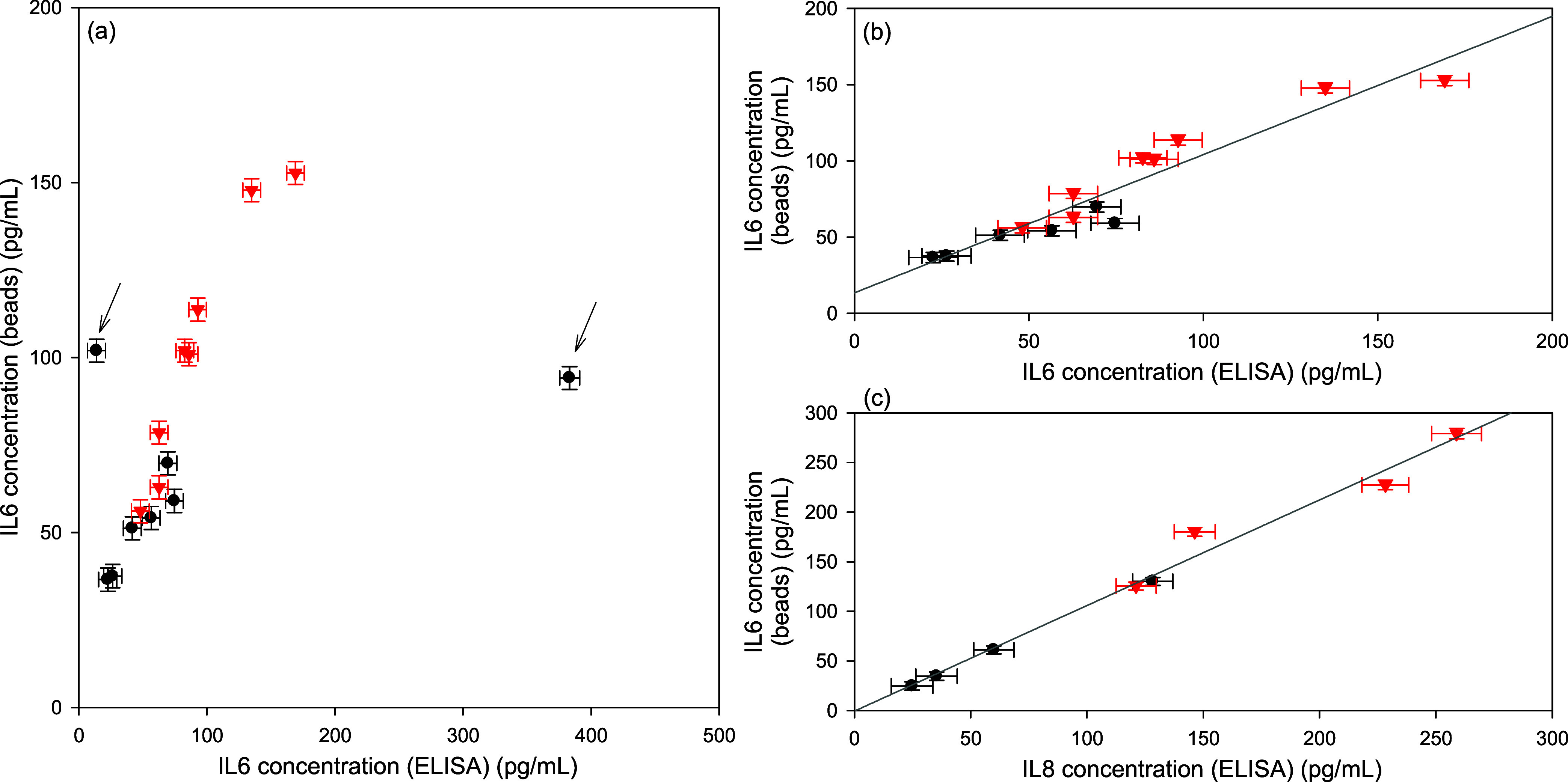
Plots of protein concentrations in saliva determined by hydrogel beads versus ELISA for (a) IL6, (b) IL6 in all samples except the two marked by arrows, and (c) IL8 (black: healthy and red: OLP, gray: best fit line, unprocessed saliva samples were used).

We hypothesized that the difference in protein concentrations determined using hydrogel beads and ELISA was because ELISA suffered from interferents in saliva. To validate this hypothesis, saliva samples were diluted by 10× and then concentration of IL6 was determined using ELISA. The determined concentrations of IL6 were multiplied by the dilution factor to obtain the protein concentration in the original saliva samples. The resulting IL6 concentrations determined by ELISA with 10× dilution were plotted against IL6 concentration determined in the same samples using hydrogel beads without sample preparation. The resulting plot is shown in Figure S11 in the Supporting Information and the slope of the best fit line was 0.99 ± 0.08 (*n* = 14 samples). Based on the *t* test, at 95% confidence, there was no significant difference in the concentrations of IL6 determined in unprocessed saliva using hydrogel beads and in 10× diluted saliva using ELISA after multiplying the obtained concentrations by the dilution factor. Thus, we concluded that the hydrogel-bead-based assay reported in this work is suited for the measurement of salivary proteins such as IL6 and IL8 without sample preparation, which contrasts with ELISA that required 10× dilution of saliva samples.

### 3.4 Stability Studies

We envision offering dry hydrogel beads to end-users (e.g., community health centers) for capture of salivary protein biomarkers. Thus, we studied the duration for which hydrogel beads can be stored dry without negatively affecting their ability to measure proteins. Furthermore, we envision that after hydrogel beads have been incubated with saliva to capture proteins, dried beads containing proteins can be sent to centralized laboratories for quantitation of proteins. Thus, we also studied the stability of the proteins captured in hydrogel beads.

- Storage stability of hydrogel beads: a batch of hydrogel beads was prepared using 5% (w:v) precursor solution. One of these freshly prepared beads was dried and used to measure IL6 in PBS. The remaining beads were stored dry and every month, one bead was used to measure IL6, and the process continued for 6 months. For the entire study, aliquots of the same stock solution of IL6 in PBS stored at −20 °C were used. The fluorescence spectra of IL6 released from hydrogel beads, which were stored for different durations, are provided in Figure S12 in the Supporting Information. The corresponding fluorescence peak intensity was plotted as a function of storage duration of dried hydrogel beads, and the resulting graph is shown in Figure 9a. Figure 9a shows that there was no change in fluorescence intensity of IL6 measured using hydrogel beads stored dry for up to 6 months. Hence, hydrogel beads can be stored dry for at least 6 months without affecting their ability to measure proteins.
- Stability of proteins captured in hydrogel beads: For this study, we incubated 4 hydrogel beads made of 20% (w:v) precursor solution in 100 μL saliva samples for 60 min with shaking. Beads were washed in PBS for 5 min. In the first instance, the following two cases were studied: (1) proteins including IL6 in a saliva sample were captured in beads and the beads containing proteins were stored dry in darkness at room temperature and (2) the same saliva sample was stored at room temperature. The concentration of IL6 was determined after storing in beads and saliva for selected time durations with data summarized in Figure 9b. Figure 9b clearly shows that the fluorescence of IL6 in saliva stored at room temperature decreased to ∼4% in 6 days. In contrast, fluorescence of IL6 captured in hydrogel beads stored at room temperature was decreased to ∼65% in 6 days. This in turn suggests that proteins stored in dry hydrogel beads are much more stable than in saliva at room temperature.
  Subsequently, we performed another study in which hydrogel beads containing proteins captured from 5 different saliva samples were immersed in either PBS or 1 M trehalose for 60 min while shaking and then stored dry at room temperature. The concentration of IL6 was determined in both cases with the data summarized in Table 5. On average, fluorescence of IL6 stored for 7 days at room temperature in beads dried after immersing in PBS and 1 M trehalose was decreased to ∼77 ± 7% (saliva samples = 5) and ∼89 ± 4% (saliva samples = 5), respectively. Based on a *t* test, there was a significant difference in the fluorescence of IL6 for beads that were immersed in buffer and 1 M trehalose before storage. Thus, the storage stability of proteins was slightly improved by immersing hydrogel beads containing proteins in 1 M trehalose before drying.

**Figure 9:**
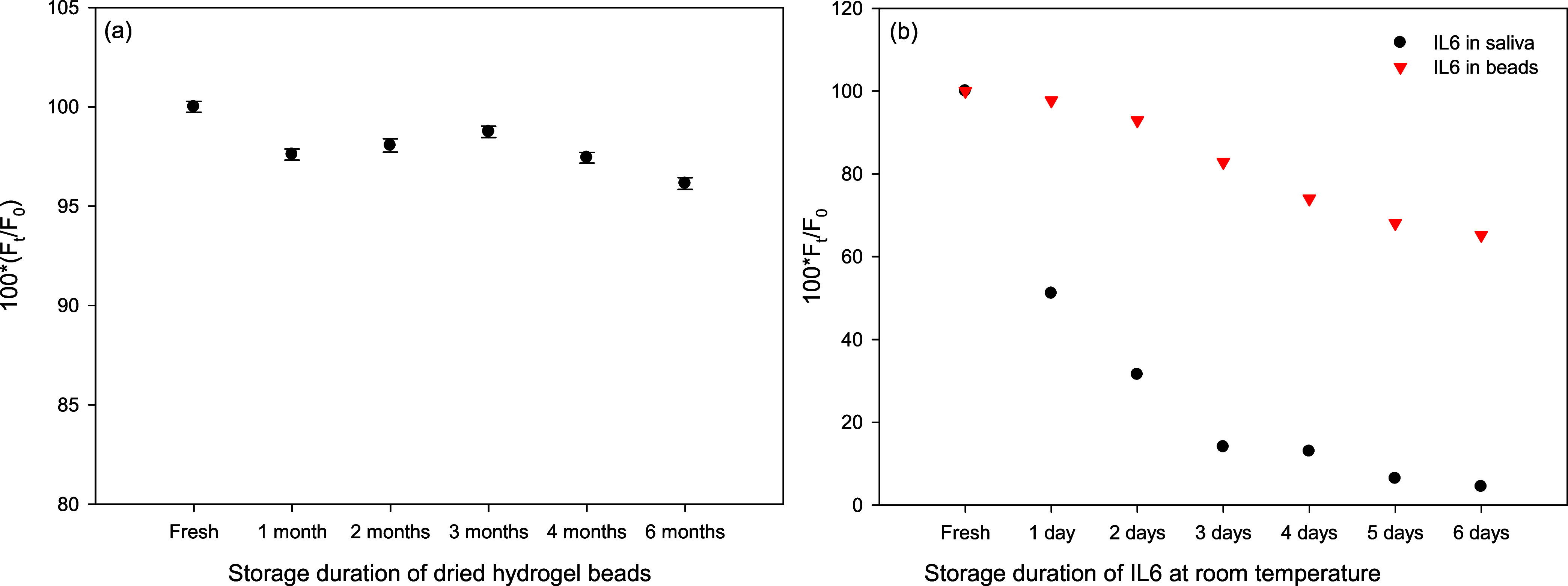
Percentage fluorescence of IL6 when (a) beads were stored dry in the dark for different durations before protein capture and (b) the protein was stored in dry beads and saliva at room temperature versus storage durations (*F*_0_ and *F*_*t*_ are fluorescence at peak wavelength for a fresh bead and that stored for duration ‘*t*’, respectively).

**Table 5:** Percentage Fluorescence of IL6 Released from 20% (w/v) Hydrogel Beads When the Beads Were Immersed in Either Buffer or 1 M Trehalose and Then Stored Dry in the Dark (*F*_0_ and *F*_*t*_ Are Fluorescence at Peak Wavelength for a Fresh Bead and That Stored for Duration ‘*t*’, Respectively, and ‘*t*’ Was Varied from Fresh to 7 Days)

Storage duration	100*(F*t*/F_0_)									
	Beads immersed in buffer after protein capture then stored dry					Beads immersed in 1 M trehalose after protein capture then stored dry				
	Saliva 1	Saliva 2	Saliva 3	Saliva 4	Saliva 5	Saliva 1	Saliva 2	Saliva 3	Saliva 4	Saliva 5
Fresh	100	100	100	100	100	100	100	100	100	100
3 days	96	93	96	91	91	96	94	99	94	89
7 days	81	75	86	68	74	92	91	92	87	83

## 4 Conclusions

This work reports hydrogel beads formed by a strain-promoted azide–alkyne click chemical reaction between a poly­(ethylene) glycol (PEG)-based cross-linker and monomers. The active monomer comprised FITC attached to a photocleavable carbonate-nitroveratryl group, which was, in turn, permanently tethered to the PEG hydrogel. The isothiocyanate group captured proteins via their surface amines, while the photocleavable group allowed captured proteins along with the attached fluorescein to be released on demand by illumination with 365 nm UV. By releasing the proteins into a smaller volume than the original sample, preconcentration of the labeled proteins was achieved. Thus, hydrogel beads were formed that could preconcentrate and fluorescently label proteins, allowing their selective capture by binding to immobilized antibodies, followed by their quantitation by fluorescence measurement.

The overall assay time, which was the sum of incubation, release, and binding times, was ∼100 min. The detection sensitivity and limit of detection was influenced by factors such as number of hydrogel beads, choice of antibodies, and sample volume; all were optimized in this work. The effect of sample viscosity on protein measurement was alleviated by increasing the incubation time. Equally, hydrogel beads acted as molecular weight cutoff filters to remove high-molecular-weight interferent proteins. The molecular weight cutoff of the beads was tuned by changing the total weight to volume of hydrogel and/or molecular weight of the inactive monomer.

We showed the utility of hydrogel beads for the measurement of interleukin 6 (IL6) and interleukin 8 (IL8) at pg/mL levels in 200 μL of minimally stimulated saliva samples of oral lichen planus (OLP) and healthy individuals. Equally, the same proteins were measured in the same saliva samples using ELISA. The head-to-head comparison with ELISA showed that hydrogel beads did not suffer from interferents in saliva and could cope with unprocessed saliva, and measurements were completed in a shorter time. We envision offering dry hydrogel beads to end-users for preconcentration of salivary protein biomarkers and removal of interferents. Afterward, protein containing dry hydrogel beads can be posted to central laboratories for analysis. With this in mind, we showed that hydrogel beads can be stored dry in darkness for at least 6 months, and proteins captured in hydrogel beads stored dry and at room temperature were significantly more stable than in saliva under the same conditions.

## Supplementary Material



## Notes

Raw fluorescence spectra and data derived from those spectra, viscosity data for PEG solutions, percentage protein recovery for different hydrogel compositions and protein molecular weights, and calibration curves of proteins obtained using hydrogel beads and ELISA (PDF)
