# Supplementary material for: Hydrogel Bead-Based Assay for the Measurement of Protein Biomarkers in Saliva
Source: ACS Meas Sci Au. 2026 May 2;6(3):837–49. doi: 10.1021/acsmeasuresciau.6c00075 (PMC13281189; doi:10.1021/acsmeasuresciau.6c00075)
Supplement: Supplementary file 1 [file tg6c00075_si_001.pdf]

Hydrogel Beads-based Assay for the Measurement of Protein Biomarkers in Saliva

Khalid Haliru[1], Nicholas J Goddard[2], Melissa Grant[3], Ana Poveda[3], Ruchi Gupta[1,]*
[1] School of Chemistry, University of Birmingham, Birmingham, B15 2TT, UK
[2] Independent researcher
[3] School of Dentistry, University of Birmingham, Birmingham, B15 2TT, UK
*Corresponding author: r.gupta.3@bham.ac.uk

Protein release kinetics

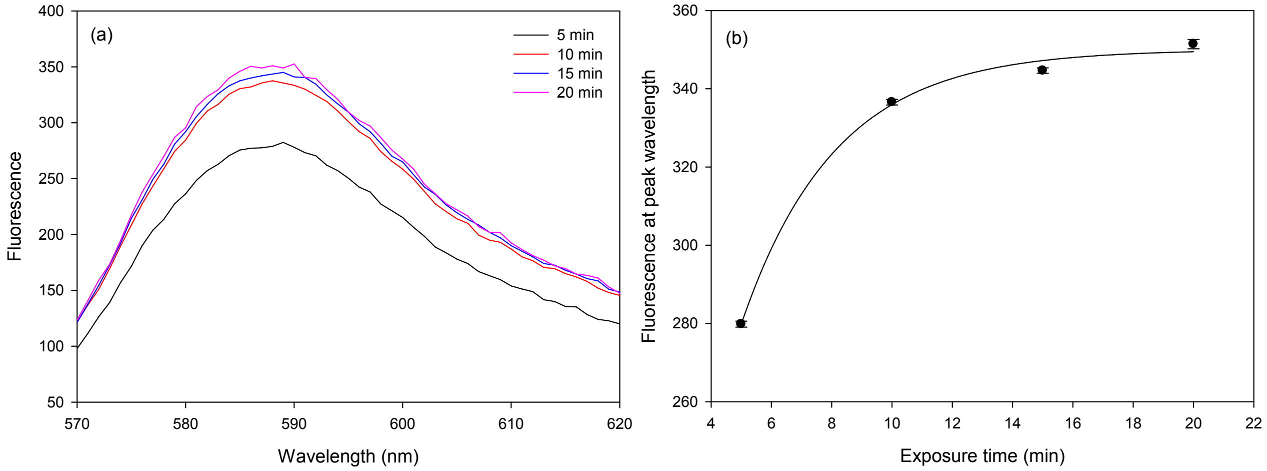

Figure S1: (a) Fluorescence spectra of rhodamine labelled streptavidin (excitation wavelength was set to 540 nm) released from 5% (w:v) hydrogel for different exposure times to 365 nm light and (b) fluorescence at peak wavelength *versus* exposure time

Immersion in amine-containing buffer after protein capture and before release

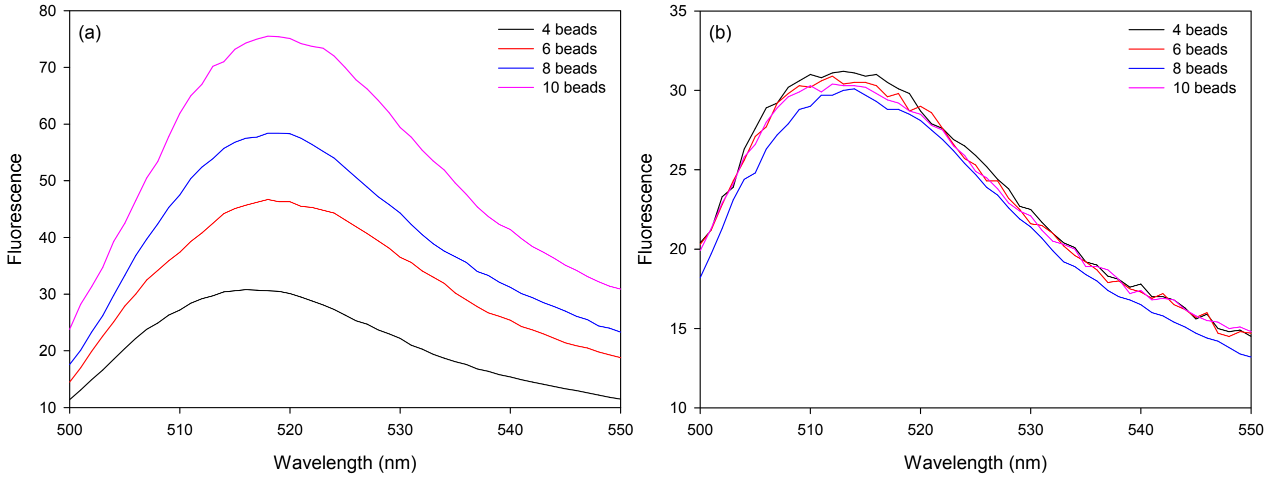

Figure S2: Fluorescence spectra of IL6 (excitation wavelength was set to 490 nm) released from 5% (w:v) hydrogel beads when 0.1% ethanolamine was (a) absent and (b) present in the buffer used to immerse hydrogel beads after protein capture and before release

Sample volume

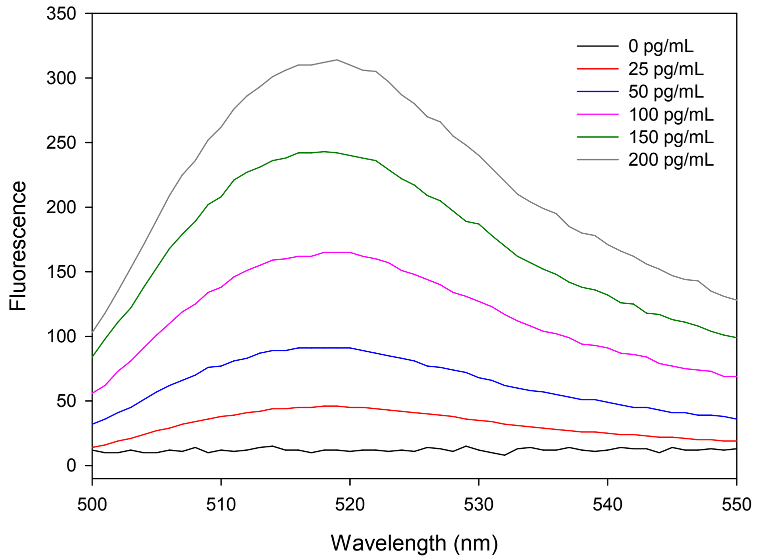

Figure S3: Fluorescence spectra of IL6 (excitation wavelength was set to 490 nm) released from 5% (w:v) hydrogel beads for 200 µL sample volume where 4 beads were incubated with different concentrations of the protein prepared in PBS

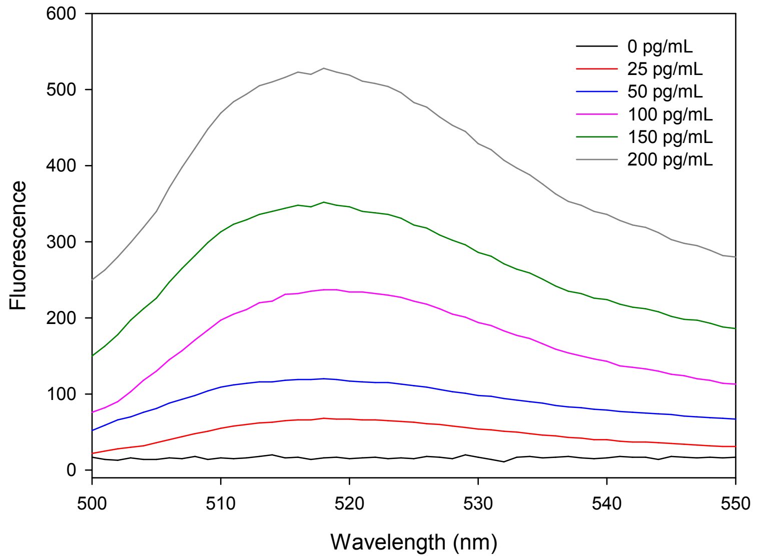

Figure S4: Fluorescence spectra of IL6 (excitation wavelength was set to 490 nm) released from 5% (w:v) hydrogel beads for 500 µL sample volume where 4 beads were incubated with different concentrations of the protein prepared in PBS

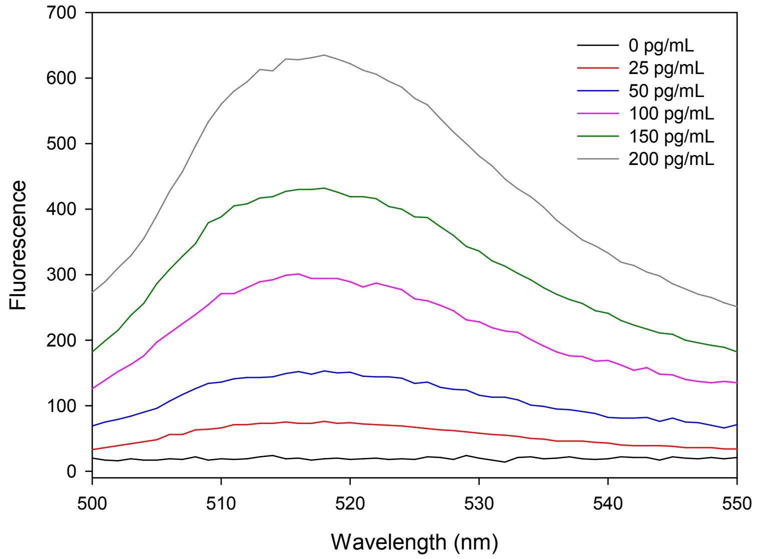

Figure S5: Fluorescence spectra of IL6 (excitation wavelength was set to 490 nm) released from 5% (w:v) hydrogel beads for 1000 µL sample volume where 4 beads were incubated with different concentrations of the protein prepared in PBS

Sample viscosity

Table S1: Summary of viscosity of different concentrations of 300,000 g/mol PEG solutions prepared in PBS buffer

Concentration of 300 kDa PEG in PBS (% w:v)	Viscosity (mPa.s)
0	1
1	10.5
2.5	36.2
5	95
7	High (could not be measured by the viscometer)

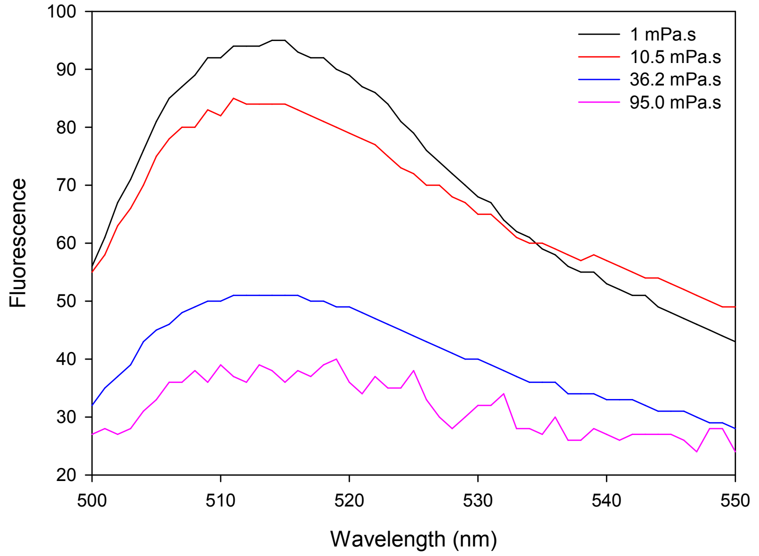

Figure S6: Fluorescence spectra of IL6 (excitation wavelength was set to 490 nm) released from 5% (w:v) hydrogel beads for 200 µL sample volume where 4 beads were incubated with 50 pg/mL IL6 prepared in 300,000 g/mol PEG solutions of different viscosity

Exclusion of chemical (protein) interferents

Table S2: Summary of percentage recovery of different molecular weight proteins for beads of different total weight to volume of hydrogel where the molecular weight of PEG bis-azide was 2,066 g/mol

Protein	Molecular weight (g/mol)	Percentage recovery for proteins for the following total weight to volume of hydrogel				
		5% (w:v)	10% (w:v)	20% (w:v)	30% (w:v)	40% (w:v)
Cytochrome C	12,000	91.9	96	77.2	73.2	69.1
Lysozyme	15,000	92.7	95	75.5	71.6	66.3
Streptavidin	55,000	95	94.4	22.2	16.2	3.6
Bovine Serum Albumin	67,000	86.8	79.4	22.1	10.3	1.3
Lactoferrin	80,000	94.8	95.5	5.8	3.9	1.8
C reactive protein	120,000	96.3	82.9	1.1	1	0.9

Table S3: Summary of percentage recovery of different molecular weight proteins for beads of different total weight to volume of hydrogel where the molecular weight of PEG bis-azide was 6,066 g/mol

Protein	Molecular weight (g/mol)	Percentage recovery for proteins for the following total weight to volume of hydrogel				
		5% (w:v)	10% (w:v)	20% (w:v)	30% (w:v)	40% (w:v)
Cytochrome C	12,000	93.3	93.3	79.2	79.9	73.2
Lysozyme	15,000	89.3	96.2	77	73.6	69.7
Streptavidin	55,000	96.4	95	25.8	18.5	5
Bovine Serum Albumin	67,000	82.4	80.9	25	13.2	2.1
Lactoferrin	80,000	96.1	96.8	7.8	5.2	1.9
C reactive protein	120,000	96.3	84.1	1.5	1.1	1.2

Table S4: Summary of percentage recovery of different molecular weight proteins for beads of different total weight to volume of hydrogel where the molecular weight of PEG bis-azide was 10,066 g/mol

Protein	Molecular weight (g/mol)	Percentage recovery for proteins for the following total weight to volume of hydrogel				
		5% (w:v)	10% (w:v)	20% (w:v)	30% (w:v)	40% (w:v)
Cytochrome C	12,000	95.3	95.3	81.2	82.6	75.8
Lysozyme	15,000	87.7	96.9	81.6	75.1	71.6
Streptavidin	55,000	94	96.4	32.5	19.5	7
Bovine Serum Albumin	67,000	85.3	86.8	30.9	16.2	2.4
Lactoferrin	80,000	96.8	96.1	12.3	6.5	2.1
C reactive protein	120,000	97.6	87.8	2.8	1.5	1.6

Table S5: Summary of percentage recovery of different molecular weight proteins for beads of different total weight to volume of hydrogel where the molecular weight of PEG bis-azide was 20,066 g/mol

Protein	Molecular weight (g/mol)	Percentage recovery for proteins for the following total weight to volume of hydrogel				
		5% (w:v)	10% (w:v)	20% (w:v)	30% (w:v)	40% (w:v)
Cytochrome C	12,000	94.6	96.6	83.2	84.6	78.5
Lysozyme	15,000	90	98.1	84.7	76.2	73.6
Streptavidin	55,000	97	97.4	36.1	20.5	8.9
Bovine Serum Albumin	67,000	89.7	85.3	35.3	19.1	3.4
Lactoferrin	80,000	98.7	94.8	16.9	8.4	2.3
C reactive protein	120,000	96.3	91.5	3.3	1.7	2

Measurement of cytokines in saliva

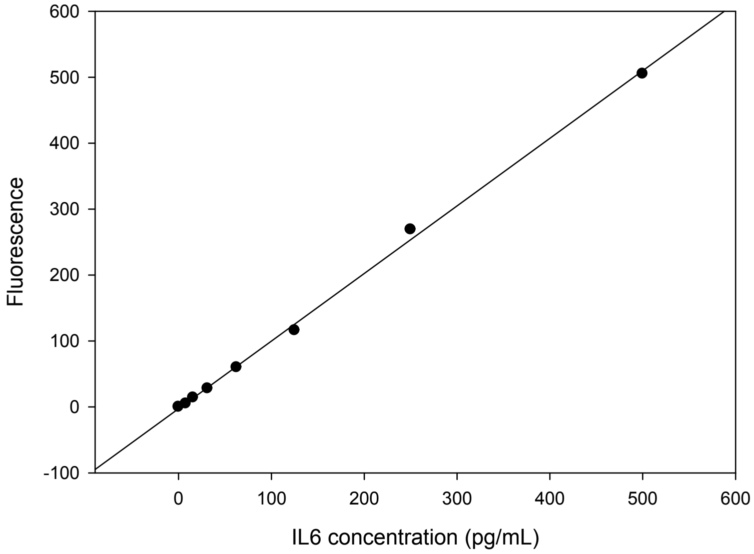

Figure S7: Calibration curve of IL6 in buffer using hydrogel beads where 4 beads of 20% (w:v) were used and sample volume was 200 µL

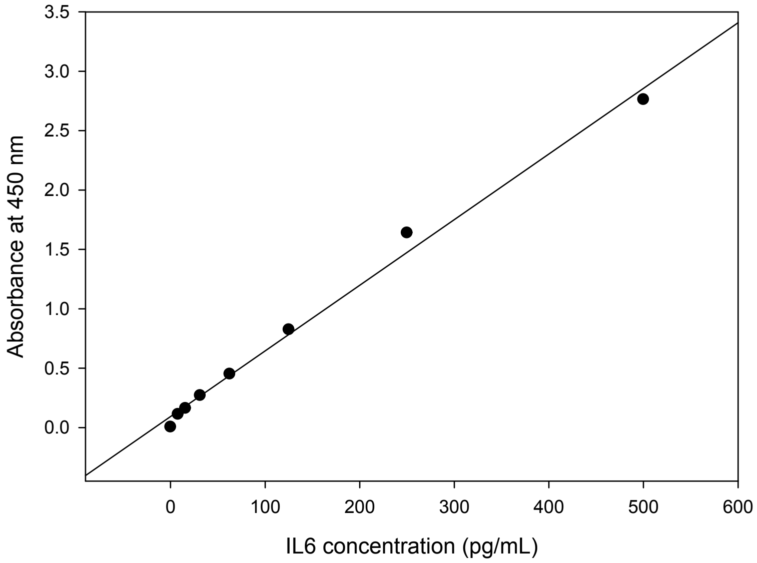

Figure S8: Calibration curve of IL6 in buffer using SimpleStep ELISA kit where sample volume was 50 µL

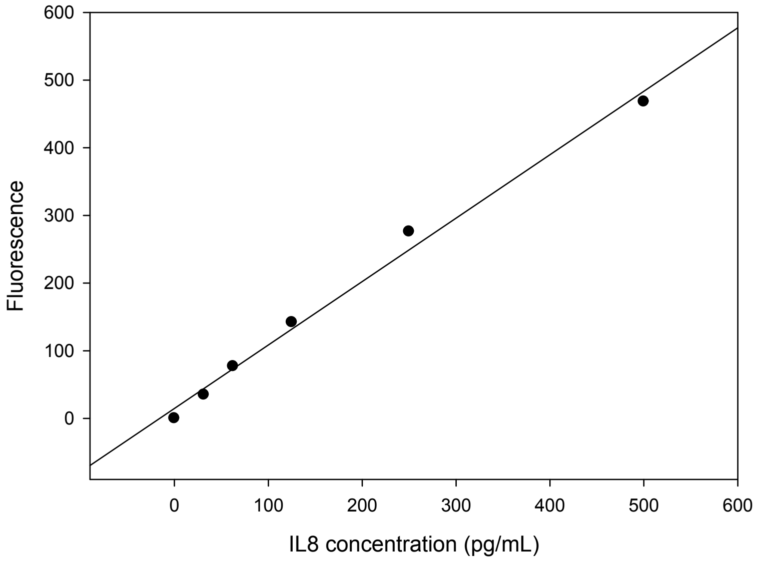

Figure S9: Calibration curve of IL8 in buffer using hydrogel beads where 4 beads of 20% (w:v) were used and sample volume was 200 µL

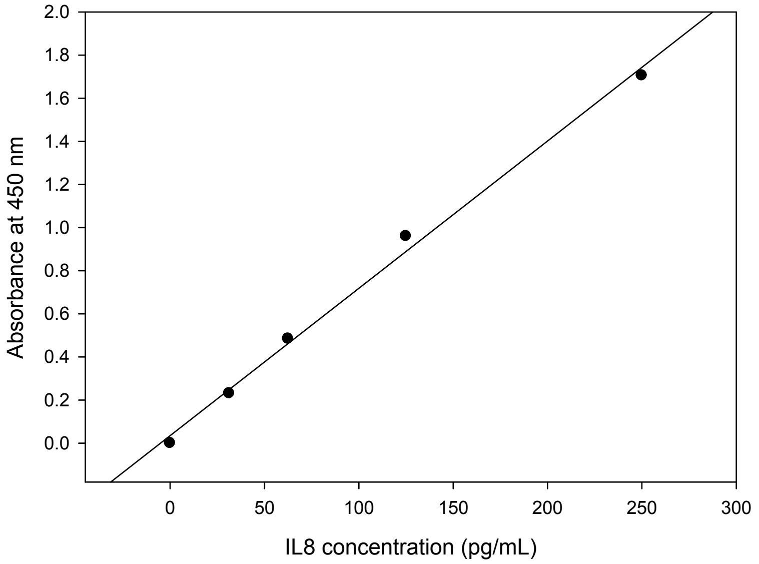

Figure S10: Calibration curve of IL8 in buffer using DuoSet ELISA kit where sample volume was 50 µL

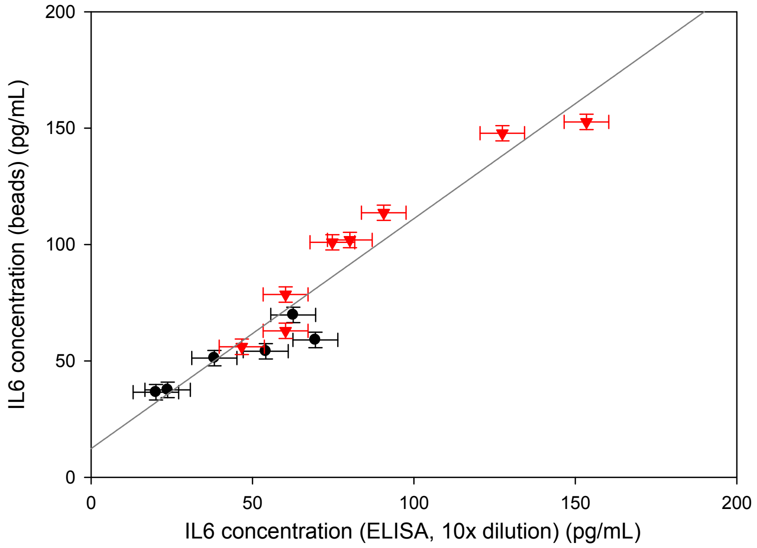

Figure S11: Plot of IL6 concentrations in saliva determined by hydrogel beads (no sample preparation) *versus* ELISA (10×dilution followed by multiplication with the dilution factor) (black: healthy and red: OLP, grey: best fit line)

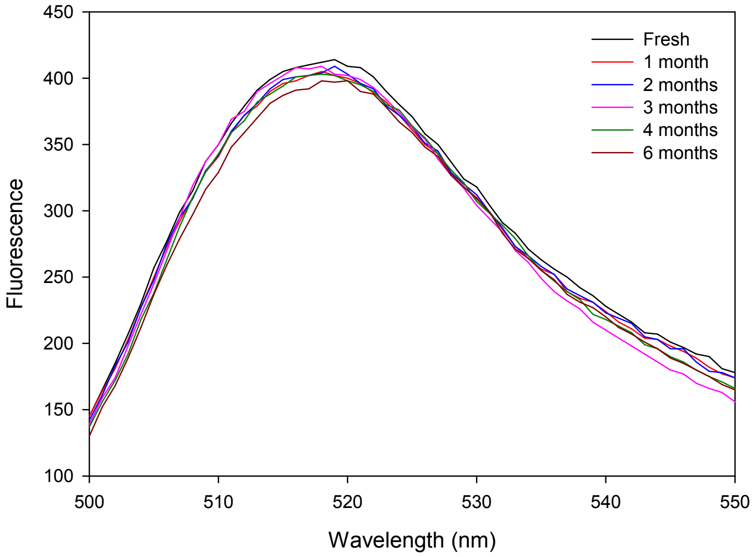

Figure S12: Plot of IL6 concentrations determined using ELISA with centrifuged saliva samples *versus* hydrogel beads used for unprocessed saliva samples (black: healthy individuals, red: OLP individuals and blue: not included in finding the best fit line)
